# Functional Impact of Post-exercise Cooling and Heating on Recovery and Training Adaptations: Application to Resistance, Endurance, and Sprint Exercise

**DOI:** 10.1186/s40798-022-00428-9

**Published:** 2022-03-07

**Authors:** Thomas Chaillou, Viktorija Treigyte, Sarah Mosely, Marius Brazaitis, Tomas Venckunas, Arthur J. Cheng

**Affiliations:** 1 School of Health Sciences, Örebro University, 701 82 Örebro, Sweden; 2 Sports Science and Innovation Institute, Lithuanian Sports University, 44221 Kaunas, Lithuania; 3 Muscle Health Research Centre, School of Kinesiology and Health Sciences, Faculty of Health, York University, Toronto, M3J 1P3 Canada

**Keywords:** Cooling, Heating, Water immersion, Training, Muscle function, Physical performance, Temperature, Fatigue, Recovery

## Abstract

The application of post-exercise cooling (e.g., cold water immersion) and post-exercise heating has become a popular intervention which is assumed to increase functional recovery and may improve chronic training adaptations. However, the effectiveness of such post-exercise temperature manipulations remains uncertain. The aim of this comprehensive review was to analyze the effects of post-exercise cooling and post-exercise heating on neuromuscular function (maximal strength and power), fatigue resistance, exercise performance, and training adaptations. We focused on three exercise types (resistance, endurance and sprint exercises) and included studies investigating (1) the early recovery phase, (2) the late recovery phase, and (3) repeated application of the treatment. We identified that the primary benefit of cooling was in the early recovery phase (< 1 h post-exercise) in improving fatigue resistance in hot ambient conditions following endurance exercise and possibly enhancing the recovery of maximal strength following resistance exercise. The primary negative impact of cooling was with chronic exposure which impaired strength adaptations and decreased fatigue resistance following resistance training intervention (12 weeks and 4–12 weeks, respectively). In the early recovery phase, cooling could also impair sprint performance following sprint exercise and could possibly reduce neuromuscular function immediately after endurance exercise. Generally, no benefits of acute cooling were observed during the 24–72-h recovery period following resistance and endurance exercises, while it could have some benefits on the recovery of neuromuscular function during the 24–48-h recovery period following sprint exercise. Most studies indicated that chronic cooling does not affect endurance training adaptations following 4–6 week training intervention. We identified limited data employing heating as a recovery intervention, but some indications suggest promise in its application to endurance and sprint exercise.

## Key Points

- The primary benefit of cooling is observed during the early recovery phase (< 1 h post-exercise): it can improve fatigue resistance after an initial endurance exercise performed in hot ambient conditions and could possibly enhance the recovery of maximal strength following resistance exercise.
- Repeated post-exercise cooling appears to blunt muscle strength adaptations and decrease fatigue resistance following resistance training (12 weeks and 4–12 weeks, respectively). In the early recovery phase, cooling could also impair sprint performance when executed < 1 h following initial sprint exercises, and some studies indicate that it could possibly reduce neuromuscular function immediately after endurance exercise.
- Single application of cooling has generally no effect during the 24–72-h recovery period following resistance and endurance exercises, while it could have some benefits on the recovery of neuromuscular function during the 24–48-h recovery period following sprint exercise. Repeated exposures to cooling do not seem to affect endurance training adaptations following 4–6-week training interventions. To date, there are a limited number of studies employing heating as a recovery intervention, but some indications suggest promise in its application to endurance and sprint exercise.

## Introduction

Various forms of exercise exist and are positioned on a continuum, with endurance exercise on one side, resistance exercise on the other side, and sprint exercise somewhere in-between. Most sporting events and exercise training sessions include one or a combination of these exercise forms. Accelerating acute recovery of neuromuscular function and physical performance after exercise is crucial for enhancing the quality of subsequent training sessions or maximizing athletic performance in multi-day competitions. In addition, improving physical recovery is beneficial for increasing the total training volume in trained athletes, which would enhance training adaptations while potentially avoiding overtraining and injury. Applying additional physiological stresses during post-exercise recovery could also be relevant for enhancing training adaptations [1].

The use of cooling such as cold-water immersion (CWI) has become a popular post-exercise recovery intervention based on the assumption that it can enhance the restoration of physical performance and augment chronic adaptations to training. Cooling has generally been thought to improve recovery by reducing the feelings of muscle soreness, alleviating exercise-induced muscle damage, and decreasing inflammation and edema, as has been discussed in previous reviews [2–6]. Application of local heating is more commonly used in the rehabilitation setting to treat musculoskeletal injuries or to protect muscle from potential damage [7, 8]. Although frequently used by athletes, its impact on post-exercise recovery and performance has not been extensively investigated [3, 7].

Numerous reviews have analyzed the potential effects of cooling [2–6, 9–11] or heating (combined or not with cooling) [2, 3, 5, 7, 10] on post-exercise recovery and training adaptations. In the current review, we postulate that cooling or heating could improve or worsen post-exercise recovery and training adaptations depending on the form of exercise (endurance, resistance or sprint exercise) given that the mechanism of exercise-induced fatigue, and thus the potential recovery mechanisms involved, are known to be task-dependent [12, 13]. Furthermore, the most relevant measure of performance outcome relates to neuromuscular function, as determined by maximal strength and power. It was recently acknowledged that there is a relative lack of research studies in this field that focus on strength and power assessment [14], and determining the effectiveness of cooling and/or heating on functional outcome measures has also not been the main focus of recent reviews exploring the role of post-exercise cooling or heating [3, 4, 7]. Thus, the current review will delineate the available evidence as to whether post-exercise cooling and/or heating are detrimental or beneficial for the recovery of neuromuscular function, fatigue resistance, physical performance, and training adaptations. Our review will include research articles that studied the effects of single and repeated applications of cooling or heating following exercise (resistance, endurance or sprint exercises). These studies will be categorized into those that investigated (1) the early acute phase of recovery (up to 9.5 h following exercise: recovery time between two exercise sessions performed within the same day), (2) the late acute phase of recovery (24–72 h following exercise: recovery time between exercise sessions performed on separate days), and (3) repeated application of the treatment. We will also outline some potential mechanisms involved and will outline key points at the end of each section.

## Definition of Exercise Forms and Modes of Cooling and Heating

Resistance exercise, traditionally defined as short exercise executed at high loads with few repetitions, is the most common form of strength training [1]. Studies involving purely eccentric exercises were not included in this category because these exercises cause severe muscle damage and represent an extreme situation that is less functionally relevant to traditional strength training regimes. Acute resistance exercise typically entailed a total of 4–16 sets at 8–20 repetitions involving a single form of exercise or a combination of exercises, and training interventions corresponded to 4–12 weeks of resistance training (2–3 sessions per week including 3–6 sets at 8–20 repetitions for each exercise) (Table 1).

**Table 1:** Effects of cooling and heating following resistance exercise on functional recovery and training adaptations

References	Participants	Exercise protocol	Post-exercise recovery method	Main finding	Main effect
*Single post-exercise exposure*					
Argus et al. [33]	Recreationally trained subjects (13 M, 26 Y)	3 × 5 deadlifts at 6 RM load + 3 × 10 back squats, bench presses, barbell lunges, and barbell bent-over rows at 11 RM load	Crossover design: Immersion up to the neck: CWI: 15 °C for 14 min. CWT: 1 min at 38 °C and 1 min at 38 °C for 14 min. CON: 20 min PR (*23 °C*)	Similar MVIC KE torque and jump performance (CMJ) in the 3 conditions **@ 5 min, 2 h and 4 h** post-recovery	Ø of CWI and CWT on neuromuscular function **@ 5 min to 4 h**
Gonzalez et al. [36]	Recreationally trained subjects (40 M, 22 Y)	4x ~ 10 squats, deadlifts, and barbell split squats at 70–80% 1RM	2 groups*: CWI: 10–12 °C for 10 min CON: 10 min PR. *exclusion of two groups (nutrition supplementation with/without CWI)	Similar number of reps and average power at 80% 1RM (squat) over four sets in CWI and CON groups **@ 24–48 h** post-Ex	Ø of CWI on fatigue resistance **@ 24–48 h**
Jajtner et al. [37]	Recreationally trained subjects (30 M)	4x ~ 10 squats, deadlifts, and barbell split squats at 70–80% 1RM	2 groups*: CWI: 10–12 °C for 10 min. CON: 10 min PR. *exclusion of one group (neuromuscular electrical stimulation)	Similar number of reps and average power at 80% 1RM (squat) over four sets in CWI and CON groups **@ 24–48 h** post-Ex	Ø of CWI on fatigue resistance **@ 24–48 h**
Pointon et al. [20]	Recreationally trained subjects (10 M, 21 Y)	6 × 25 maximal CONC (60°/s)/ECC (120°/s) single leg isokinetic KE. *20 °C*	Crossover design: CWC: ice cuff (exercised leg) for 20 min. CON: 20 min PR	Similar MVIC KE torque, potentiated twitch torque and VA in CWC and CON **@ 2, 24 and 48-h** post-recovery. Similar voluntary EMG (RMS) and M-wave amplitude in CWC and CON **@ 2, 24 and 48 h** after recovery	Ø of CWI on neuromuscular function **@ 2–48 h**
Roberts et al. [29]	Recreationally trained subjects (10 M, 21 Y)	6 × squats to failure at 8–12 RM loads. 3 × 12 walking dumbbell lunges at 40% body mass load 3 × 12 countermovement DJ *-24 °C, 49% RH*	Crossover design. CWI: 10 °C for 10 min (up to the clavicle). CON: active recovery cycling at ~ 45 W for 10 min	Similar maximal isometric squat force and jump performance (SJ, CMJ) in CWI and CON **@ 2–4 h** after Ex. Greater recovery of average and total load lifted during 6 × 10 squats at 80% 1RM in CWI versus CON **@ 6-h** post-Ex	**↑** of CWI on fatigue resistance **@ 6 h**. Ø of CWI on maximal muscle function **@ 2–4 h**
Roberts et al. [30]	Recreationally trained subjects (10 M, 21 Y)	10 × 20 maximal isokinetic concentric KE at 90°/s. *24 °C and 43.5% RH*	Crossover design: CWI: 10 °C for 10 min. CON: active recovery cycling at ~ 41 W for 10 min	Reduced MVIC KE torque **@ 5, 20 and 40 min** post-recovery in CON (vs. to pre-Ex) but not in CWI. Similar fatigue resistance (50 reps isokinetic KE at 90°/s) **@ 60 min** post-recovery in CWI and CON	**↑** of CWI on MVIC: **@ 5–40 min**. Ø of CWI on fatigue resistance **@ 60 min**
Wilson et al. [31]	Recreationally trained subjects (24 M, 25 Y)	80% 1RM: 4 × 6 back squats. 4 × 8 split squats, hip thrusts, Romanian deadlifts	2 groups: CWI: 10 °C for 10 min. PLA: 10 min PR with ingestion of a cornstarch pill (placebo). *exclusion of one group (cryotherapy chamber)	Lower recovery of MVIC KE torque **@ 24–48 h** post-Ex in CWI versus PLA groups. Lower recovery of maximal isometric squat force in CWI versus PLA groups **@ 48 h** post-Ex, with similar recovery in the 2 groups **@ 24 and 72 h** post-Ex. Lower recovery of maximal isokinetic KE torque (60°/s) **@ 24–48 h** post-Ex in CWI versus PLA groups. Lower recovery of CMJ performance **@ 48–72 h** post-Ex in CWI versus PLA groups	**↓** of CWI (vs. PLA) on maximal strength and jump performance **@24–72 h**
*Repeated post-exercise exposures*					
Fröhlich et al. [34]	Recreationally trained subjects (17 M, 23 Y)	5-wk RT (# session/wk not stated): 3 × 8–12 CONC and ECC knee flexions at 75–80% 1RM	Immersion after each session, contralateral limb-control design: CWI: 3 × 4 min at 12 °C with 30 s rest. CON (leg 2): PR	Similar increase in maximal force (1RM, KF) in CWI and CON. Lower increase in fatigue resistance (12RM, KF) in CWI versus CON	Ø of CWI on maximal strength. ↓ of CWI on fatigue resistance
Fyfe et al. [35]	Recreationally trained subjects (16 M, 25 Y)	7-wk RT (3 sessions/wk): 3 × 12-RM or 20-RM (20 upper and lower body and trunk Ex)	Immersion after each session, 2 groups: CWI (up to the sternum): 10 °C for 15 min. CON: 15 min PR	Similar increase in maximal force (1RM bench press and leg press) in both groups. Similar peak SJ force and push-up force in both groups after training. Smaller gain in peak CMJ force in CWI versus CON groups	Ø of CWI on maximal strength. Ballistic Ex: Ø (SJ and push-up) or ↓ (CMJ) of CWI
Ohnishi et al. [39]	Recreationally trained subjects (16 M, 21 Y)	6-wk RT (3 sessions/wk): 3 × 8-RM handgrip Ex	Unilateral immersion of the elbow joint and lower arm after each session. 2 groups. CWI: 10 °C for 20 min. CON: PR	No improvement of MVIC handgrip force in both CWI and CON groups. Improvement of fatigue resistance (number of reps at 30% RM with a pace of 30 reps/min) lower (tendency) in CWI versus CON	Ø of CWI on maximal strength. Potential ↓ of CWI on fatigue resistance
Poppendieck et al. [53]	Recreationally trained subjects (9 M and 2 F, 25 Y)	8-wk RT (3 sessions/wk): 3 × 10-RM (leg press, KF and KE)	Immersion after each session, crossover design (8-wk washout period): CWI (up to the neck): 14–15 °C for 10 min. CON: 10 min PR	No improvement of maximal force (1-RM leg press) and jump performance (CMJ) in both CWI and CON	Ø of CWI on maximal strength and jump performance
Roberts et al. [55]	Recreationally trained subjects (24 M, 21 Y)	12-wk RT (2 sessions/wk): 3–6 × 8–12 reps at 8–12 RM loads (leg press, KE, KF) and 3 × 10–18 reps (walking lunges, plyometrics). *23–25 °C*	Immersion after each session, 2 groups: CWI: 10 °C for 10 min. CON: 10 min active recovery cycling at ~ 60 W	Lower increase in maximal force (leg press force, KE force and MVIC KE torque) in CWI versus CON groups. No improvement of maximal isokinetic KE torque (90°/s) in both groups. Fatigue resistance (50 reps isokinetic KE at 90°/s): increase after training only in CON group over 1–25 reps	↓ of CWI on maximal force (except isokinetic torque). ↓ of CWI on fatigue resistance
Stadnyk et al. [25]	Recreationally trained subjects (5 M and 5 F, 21 Y)	12-wk RT (2–3 sessions/wk): 4 × 8 reps at 70% 1RM (ECC and CONC single limb KE)	Contralateral limb-control design: Heat (heat pad wrapped around the thigh): ~ 40 °C during and for 20 min after each session. CON: PR	Similar increase in peak and mean isokinetic torque (CONC KE at 90°/s) in heat and CON legs	Ø of Heat on muscle strength
Yamane et al. [32]	Contralateral limb-control design: Sedentary subjects (7 M + 4 F, 20 Y) 2 groups (unilateral immersion): Sedentary subjects (16 M, 21 Y)	4-wk RT (3 sessions/wk): 3 × 8 isotonic handgrip Ex at 70–80% 1RM. *25 °C, 50% RH*	Unilateral immersion of the elbow joint and lower arm after each session. Contralateral limb-control design: CWI: 10 °C for 20 min. CON: PR. 2 groups. CWI: 10 °C for 20 min. CON: PR	Similar increase in MVIC handgrip force in CWI and CON (both experiments). Improvement of fatigue resistance (number of reps at 30% RM with a pace of 30 reps/min) lower in CWI versus CON (contralateral limb-control design), or similar in CWI and CON groups (2 groups)	Ø of CWI on maximal strength. Ø or ↓ of CWI on fatigue resistance
Yamane et al. [38]	Recreationally trained subjects (14 M, 20 Y)	6-wk RT (3 sessions/wk): 5 × 8 wrist-flexion at 70–80% 1RM. *25 °C, 50% RH*	Unilateral immersion of the elbow joint and lower arm after each session. 2 groups. CWI: 10 °C for 20 min. CON: PR	Improvement of MVIC wrist flexor force lower in CWI versus CON. Improvement of fatigue resistance (number of reps at 35% RM with a pace of 30 reps/min) lower (tendency) in CWI versus CON	↓ of CWI on maximal strength and fatigue resistance

Water immersion was applied up to the waist/lower part of the trunk, unless stated otherwise. Text highlighted in italic describes the ambient condition, when stated (air temperature and relative humidity)

Text in bold describes the specific time points

*CMJ* countermovement jump, *CON* control, *CONC* concentric, *CWC* cold water cuff, *CWI* cold water immersion, *DJ* drop jump, *ECC* eccentric, *Ex* exercise, *F* female, *KE* knee extension, *KF* knee flexion, *M* male, *MVIC* maximal voluntary isometric contraction, *PLA* placebo, *PR* passive recovery, *reps* repetitions, *RH* relative humidity, *RM* repetition maximum, *RMS* root mean square, *SJ* squat jump, *RT* resistance training, *VA* voluntary activation assessed via interpolated twitch technique, *wk* week, *Y* year, ↑ positive effect, ↓ negative effect, Ø no effect

*Some groups were excluded because they were not relevant for the purpose of the review

Endurance exercise encompasses prolonged exercise performed at intensities below or close to maximal oxygen uptake (VO_2max_) that highly stimulates the cardiorespiratory system and aerobic metabolism. Prolonged exercises consisting of numerous interval bouts performed at high intensities (near VO_2max_, all-out sprints excluded) and interspaced with efforts performed at lower intensity were also included in this category. Prolonged exercise sessions combining repetitive all-out sprints and moderate/high intensity aerobic exercises were excluded from this review since a purpose of this study was to isolate the effects of treatment on specific forms of exercise [15, 16]. Acute endurance exercise usually included continuous or interval bouts of cycling or running performed for ~ 30 min up to 3–4 h (Table 2). Chronic exercise consisted of 4–5 weeks of predominantly endurance training (3–4 sessions/week up to 2.5–4 h/day) (Table 2).

**Table 2:** Effects of cooling and heating following endurance exercise on functional recovery and training adaptations

References	Participants	Exercise protocol	Post-exercise recovery method	Main finding	Main effect
*Single post-exercise exposure*					
Brophy-Williams et al. [74]	Well trained team-sport players (8 M, 21 Y)	Running: 8 × 3 min at 90% Vmax. *23 °C and 43% RH*	Crossover design: CWI: 15 °C for 15 min immediately or 3 h post-Ex. CON: 15 min PR immediately after Ex	Higher number of shuttles completed (Yo-Yo intermittent recovery test) **@ 24 h** post-Ex in CWI (immediately > 3 h post-Ex) versus CON	**↑** of CWI (immediately post-HIIE) on shuttle test performance **@ 24 h**
Chauvineau et al. [101]	Well trained runners (12 M, 28 Y)	Running: Simulated trail run for 48 min *21 °C and 44% RH*	Crossover design: CWI (whole body including the head): 13 °C for 10 min. CWI (up to the waist): 13 °C for 10 min. CON: 10 min PR (*19 °C*)	Similar recovery of MVIC KE torque and CMJ performance **@ 24–48 h** post-Ex in all conditions	Ø of CWI on maximal strength and jump performance **@ 24–48 h**
Cheng et al. [19]	Recreationally trained subjects (4 F and 1 M, 26 Y)	Arm cycling: 3 × 5 min (all-out) + 4 × 15 min at 50% VO_2max_	Crossover design. Use of water-perfused arm cuff: Ice-chilled for 2 h. Heated at 38 °C for 2 h. CON: heated at ~ 33 °C for 2 h	Better maintenance of mean PO (3 × 5 min all-out arm cycling) **immediately** after recovery method after heating versus cooling	**↑** of heating and **↓** of cooling on fatigue resistance **@ 0 h**
Crampton et al. [75]	Recreationally trained triathletes (9 M, 30 Y)	Cycling: 5 min at 50% VO_2max_ + 5 min at 60% VO_2max_ + 80% VO_2max_ until exhaustion. *21 °C*	Crossover design: CWI: 15 °C for 30 min. TWI: 34 °C for 30 min. CWT: 2,5 min at 8 °C and 2,5 min at 40 °C for 30 min. CON: 30 min active recovery	Greater time to failure during intense cycling (80% VO_2max_) **@ 5 min** post-recovery in CWI versus other conditions, and in CWT versus CON	**↑** of CWI and CWT (to a lower extent) on fatigue resistance **@ 5 min**
Dantas et al. [97]	Recreationally trained runners (30 M, 32 Y)	Running: 10-km TT run. *30 °C and 69% RH*	3 groups: CWI: 10 °C for 10 min. TWI: 30 °C for 10 min. CON: 10 min PR	Similar jump performance (triple hop distance) and strength (maximal voluntary concentric KE at 60°/s) in the 3 groups **immediately** and **@ 24 h** post-recovery	Ø of CWI on jump performance and maximal strength (**@ 0 and 24 h**
De Paula et al. [22]	Recreationally trained subjects (9 M, 24 Y)	Running: Unilateral ECC KF EX + 90 min running (70% VO_2peak_). *20 °C and 70% RH*	Crossover design: CWI: 15 °C for 15 min. HWI: 38 °C for 15 min. TWI: 28 °C for 15 min. CON: 15 min PR	Similar 5-km running time **@ 4 h** post-recovery in all conditions	Ø of CWI and HWI on endurance performance **@ 4 h**
De Pauw et al. [82]	Trained subjects (9 M, 22 Y)	Cycling: 60 min at 55% Pmax + 30 min TT (Ex1). 60 min recovery. TT to perform a work equivalent to 12 min at 85% Pmax (Ex2). *30 °C and 50% RH*	Crossover design: Immersion starting directly after Ex1: CWI (up to the sternum): 15 °C for 15 min *(30 °C).* CON: 15 min PR *(30 °C)*. *exclusion of one condition (active recovery)	Similar cycling performance of Ex2 in the 2 conditions **@ 60 min** post-Ex1. Gradual decline of PO after the onset of Ex2 in PR, but not in CWI	Ø of CWI on subsequent (**@ 60 min)** endurance performance
Dunne et al. [76]	Well trained subjects (9 M, 29 Y)	Running: 5 min at 50% Vmax + 5 min at 60% Vmax + 90% Vmax until exhaustion. *22 °C*	Crossover design: CWI: 15 °C or 8 °C for 15 min. CON: 15 min PR	Higher time to failure at 90% Vmax **@ 5 min** post-recovery in CWI (8 °C only) versus CON	**↑** of CWI (8 °C only) on fatigue resistance **@ 5 min**
McCarthy et al. [77]	Recreationally trained subjects (15 M, 21 Y)	Cycling: 12 min at 85% VT + 30 s/30 s interval bouts (90% peak PO/40% peak PO) to exhaustion. *19 °C*	Crossover design: CWI: 8 °C for 5 or 10 min. CON: PR	Higher time to failure (30 s/30 s interval bouts at 90% peak PO/40% peak PO) **immediately** post-recovery in CWI (5 and 10 min) versus CON	**↑** of CWI on fatigue resistance **@ 0 h**
Peiffer et al. [95]	Well trained cyclists (10 M, 27 Y)	Cycling: 90 min at 80% VT2 + 16.1 km TT. *32 °C and 55% RH*	Crossover design: CWI: 14.3 °C for 20 min. CON: 20 min PR *(24 °C)*	Lower MVIC and SMVIC KE torques **@ 45 and 90 min** post-Ex in CWI versus CON	**↓** of CWI on maximal strength **@ 45–90 min**
Peiffer et al. [96]	Well trained cyclists (12 M, 29 Y)	Cycling: Time-to-exhaustion test at VT1. *40 °C and 40% RH*	Crossover design. Immersion starting 25 min post-Ex: CWI: 14.3 °C for 5, 10 or 20 min. CON: 20 min PR *(24 °C)*	Similar MVIC KE torque and isokinetic KE torque (240°/s) **@ 55 min** post-Ex in CWI and CON	Ø of CWI on maximal strength **@ 55 min**
Peiffer et al. [78]	Well trained cyclists (10 M, 35 Y)	Cycling: 25 min at 65% VO_2max_ + 4-km TT. *35 °C and 40% RH*	Crossover design: Immersion starting 25 min post-Ex: CWI: 14 °C for 5 min *(35 °C).* CON: PR *(35 °C)*	Greater 4-km TT performance (performed after 25 min at 65% VO_2max_) (*35 °C*) **immediately** post-recovery in CWI versus CON	**↑** of CWI on endurance performance in heat **@ 0 h**
Rowsell et al. [85]	Well trained triathletes (7 M, 29 Y)	Running: 7 × 5 min at 105% anaerobic threshold. *21 °C and 40% RH*	Crossover design. Immersion starting 10 min post-Ex: CWI: 5 × 1 min at 10 °C, with 1 min rest. TWI: 5 × 1 min at 34 °C, 1 min rest	Similar mean PO (5-min maximal cycling effort + 6 × 5 min freely paced cycling) **@ 9.5 h** post-Ex in CWI and TWI	Ø of CWI on endurance performance **@ 9.5 h**
Stanley et al. [84]	Well trained cyclists (18 M, 27 Y)	Cycling: 60 min including 8 × 4 min at 80% peak PO. *22 °C*	Crossover design. Immersion starting 20 min after Ex, up to the neck: CWI: 14 °C for 5 min. CWT: 1 min at 14 °C and 2 min at 35 °C for 10 min. CON: 10 min PR	Similar TT performance (~ 14 min cycling) **@ 2.75 h** post-recovery in all conditions	Ø of CWI and CWT on endurance performance **@ 2.75 h**
Stenson et al. [98]	Well trained runners and triathletes (9 M, 36 Y)	Running: 8 × 1200 m at 75% VO_2peak_	Crossover design: CWI: 12 °C for 12 min. CON: 12 min PR	Similar 5-km running TT performance **@ 24 h** post-Ex in CWI and CON	Ø of CWI on endurance performance **@ 24 h.**
Vaile et al. [79]	Well trained cyclists (10 M, 32 Y)	Cycling: 15 min at 75% peak PO + 15 min TT. *34 °C and 39% RH*	Crossover design: Immersion up to the neck: CWI intermittent: 5 × 1 min at 10 °C, 15 °C or 20 °C, with 2 min rest. CWI: 20 °C for 15 min. CON: active recovery *(31 °C and 48% RH)*	Reduced cycling performance (15 min at 75% peak PO + 15 min TT in heat: *34 °C and 39% RH*) **@ 40 min** post-recovery in CON but not in CWI conditions. No differences between the CWI conditions	**↑** of CWI on endurance performance in hot environment **@ 40 min**
Vaile et al. [80]	Well trained cyclists (10 M, 34 Y)	Cycling: 15 min at 75% peak PO + 15 min TT. *33 °C and 44% RH*	Crossover design: Immersion up to the neck: CWI intermittent: 5 × 1 min at 10 °C, 15 °C or 20 °C, with 2 min rest. CWI: 20 °C for 15 min. CON: active recovery *(33 °C and 44% RH)*	Reduced cycling performance (15 min at 75% peak PO + 15 min TT in heat: *33 °C and 44% RH*) **@ 40 min** post-recovery in CON but not in CWI conditions. No differences between the CWI conditions	**↑** of CWI on endurance performance in hot environment **@ 40 min**
Versey et al. [87]	Well trained cyclists (11 M, 32 Y)	Cycling: 75 min including 6 sets of 5 × 15 s sprints interspaced with 15–45 s rest + 3 × 5 min TT (Ex1). 2-h recovery. Ex2: same as Ex1. *23 °C and 44% RH*	Crossover design. Immersion up to the neck, starting 10 min after Ex1: CWT: 1 min at 38 °C and 1 min at 15 °C for 6, 12 or 18 min. CON: 20 min PR (*24 °C and 48% RH*)	Greater cycling TT performance and cycling sprint performance (total work) in CWT (6 min) versus CON. Greater cycling sprint performance (total work and peak power) in CWT (12 min) versus CON	**↑** of CWT (up to 12 min) on subsequent (**@ 2 h**) cycling performance
Versey et al. [88]	Well trained runners (10 M, 37 Y)	Running: 15-min warm-up including 3 × 100 m + 3-km TT + 8 × 400 m + 7-min warm-down (Ex1) 2-h recovery. 15-min warm-up including 3 × 100 m + 3-km TT + 7-min warm-down (Ex2). *15 °C and 50% RH*	Crossover design. Immersion up to the neck, starting 10 min after Ex1: CWT: 1 min at 38 °C and 1 min at 15 °C for 6, 12 or 18 min. CON: 20 min PR (*22 °C and 43% RH*)	Slightly faster 3-km running TT of Ex2 in CWT (6 min only) versus CON	Slight **↑** of CWT (6 min) on subsequent (**@ 2 h**) endurance performance
Wilson et al. [100]	Recreationally trained runners (31 M, 40 Y)	Running: Competitive marathon (42,2 km)	2 groups: CWI: 8 °C for 10 min. PLA: PR with ingestion of fruit flavored drink (placebo). *exclusion of one group (cryotherapy chamber)	Similar recovery of peak isokinetic KE torque (60°/s), MVIC KE force and DJ performance (reactive strength index) **@ 24–48 h** post-marathon in CWI and PLA	Ø of CWI on maximal force and jump performance **@ 24–48 h**
Wiewelhove et al. [94]	Recreational runners (46 M, 30 Y)	Running: Competitive half-marathon (21,1 km)	2 groups: CWI: 15 °C for 15 min. CON: 15 min PR. *exclusion of two groups (active recovery and massage therapy)	Moderate harmful effect of CWI versus CON on CMJ performance **immediately** post-recovery, but no effects **@ 24 h**	Potential **↓** of CWI on jump performance **@ 0 h** but not **@ 24 h**
Yeargin et al. [81]	Well-trained runners (12 M and 3 F, 28 Y)	Running: 90 min of moderately intense running. *27 °C*	Crossover design: CWI: 14 °C or 5 °C for 12 min *(29 °C).* CON: PR in hot air condition *(29 °C)*	Greater 3.2-km TT running performance **@ 15 min** post-recovery in CWI (14 °C only) versus CON	**↑** of CWI on endurance performance in heat **@ 15 min**
*Repeated post-exercise exposures*					
Aguiar et al. [104]	Recreationally trained subjects (17 M, 23 Y)	4-wk endurance cycling training (12 HIIE sessions): 8–13 × 60 s at 90–110% peak PO with 75 s rest	Immersion after each training session, 2 groups: CWI: 10 °C for 15 min. CON: PR	Similar improvement of 15 km- cycling TT performance in CWI and CON groups	Ø of CWI on the improvement of endurance performance
Halson et al. [105]	Well trained subjects (21 M, 20 Y)	39 day- endurance cycling training (1–2 sessions/day): Low-moderate intensity road rides + HIIE sessions	Immersion 4x /wk, 2 groups: CWI: 15 °C for 15 min (up to the neck). CON: PR	Unclear greater increase in 2 × 4-min maximal cycling effort in CWI versus CON groups. Likely higher fatigue resistance (mean power of 2nd versus 1st 4-min maximal effort) in CWI versus CON groups. Likely greater increase in mean sprint PO in CWI versus CON groups. No between-group difference in 10 min TT performance	Unclear or likely **↑** of CWI on endurance/sprint performance
Méline et al. [23]	Elite short-track speed skaters (3 F and 3 M, 21 Y)	4-wk training (18 h/wk): ice skating, running, cycling, roller skating, fitness, RE)	Immersion after the last session of the day, crossover design. HWI: 40 °C for 20 min. CON: 20 min PR (*20 °C and 70% RH*)	MVIC KE force increased in HWI and decreased in PR. No effect of training on SJ/CMJ performance, sprint peak PO, 1-min continuous CMJ, VT and time to exhaustion during incremental test in HWI and PR. Tendency to increased VO_2max_ only in HWI. Increase ice-skating sprint performance similar in HWI and PR	↑ of HWI on maximal strength and VO_2max_ (tendency). Ø of HWI on other aerobic and anaerobic parameters
Vaile et al. [16]	Well trained cyclists (12 M, 32 Y)	5 consecutive training days: 105 min cycling (sprints + TT)	Immersion up to the neck after each session, crossover design: CWI: 15 °C for 14 min. HWI: 38 °C for 14 min. CWT: 1 min at 15 °C and 1 min at 38 °C for 14 min. CON: PR	Greater sprint performance in CWI and CWT versus CON from the 4th day, no effect of HWI. Greater TT performance in HWI (only on day 2), CWI and CWT versus CON	↑ of CWI and CWT on maintenance of high-intensity cycling performance
Yamane et al. [32]	Recreationally trained subjects (6 M, 20 Y)	4-wk cycling training (3–4 sessions/wk) 5 min at 35% VO_2max_ + 25 min at 70% VO_2max_. *25 °C and 50% RH*	Unilateral immersion after each session: CWI: 2 × 20 min at 5 °C (thigh and lower leg) with 30 min rest. CON: PR	Improvement of endurance performance (time of the one-leg incremental test) and VO_2max_ only in the CON leg	**↓** of CWI on endurance-training adaptation
Zurawlew et al. [24]	Recreationally trained subjects (17 M, 23 Y)	6 consecutive training days: 40 min running at 65% VO_2max_. *18 °C and 40% RH*	Immersion after each session, 2 groups: HWI group: 40 °C for 40 min. TWI group: 34 °C for 40 min	Reduced 5-km running time performed in heat (*33 °C, 40% RH*) after training in HWI only. No changes in 5-km running time performed in temperate environment (*18 °C, 40% RH*) after training in both groups	**↑** of HWI on endurance performance in heat

Water immersion was applied up to the waist/lower part of the trunk, unless stated otherwise. Text highlighted in italic describes the ambient condition, when stated (air temperature and relative humidity)

Text in bold describes the specific time points

*CMJ* countermovement jump, *CON* control, *CWI* cold water immersion, *CWT* contrast water therapy, *DJ* drop jump, *ECC* eccentric, *Ex* exercise, *F* female, *HIIE* high intensity interval exercise, *HWI* hot water immersion, *M* male, *KE* knee extension, *KF* knee flexion, *MVIC* maximal voluntary isometric contraction, *PO* power output, *PR* passive recovery, *SMVIC* maximal voluntary isometric contraction with superimposed electrical stimulation, *RE* resistance exercise, *RH* relative humidity, *TT* time trial, *TWI* thermoneutral water immersion, *Vmax/Pmax*: maximal speed/power obtained during a maximal incremental test (VO_2max_ test), *VO*_*2*_ oxygen uptake, *VT* ventilatory threshold, *wk* week, ↑ positive effect, Y year, ↓ negative effect, Ø no effect

*Some groups/conditions were excluded because they were not relevant for the purpose of the review

Sprint exercise included a few bouts (2–12) of all-out sprints (from ~ 15 s up to ~ 90 s) interspersed with prolonged recovery periods (at least 8 times the duration of the sprint) (Table 3). Only one study investigated chronic adaptations to sprint training (3 cycling sessions/week for 6 weeks) when combined with post-exercise CWI [17] (Table 3). The physical and physiological demands of team-sports activities require the repetition of high to maximal efforts such as sprints, jumps, kicks and tackles (i.e., sprint exercises and strength exercises) interspersed with efforts at low/moderate intensity (i.e., endurance exercise) [18]. Thus, exercise-induced fatigue in team sports is likely the result of numerous confounding factors. Although the literature related to post-exercise cooling is rather extensive in team sports [5], we purposely did not include this topic in the current review since the purpose here is to isolate the effects of treatment based on specific forms of exercise.

**Table 3:** Effects of cooling and heating following sprint exercise on functional recovery and training adaptations

References	Participants	Exercise protocol	Post-exercise recovery method	Main finding	Main effect
*Single post-exercise exposure*					
Broatch et al. [120]	Recreationally trained subjects (30 M, 24 Y)	Cycling: 4 × 30 s all-out with 4 min rest	3 groups: CWI: 10.3 °C for 15 min. TWI: 34.7 °C for 15 min. TWP: 34.7 °C for 15 min with skin cleanser added to the water (placebo)	Greater peak MVIC KE torque **immediately** post-recovery, and **@ 1 h and 48 h** post-Ex in TWP versus TWI groups, with no differences in CWI versus TWI and TWP groups **immediately** post-recovery, and **@ 1, 24 and 48 h** post-Ex. Greater average MVIC KE torque **immediately** post-recovery, and **@ 1, 24 and 48 h** post-Ex in TWP versus TWI groups, and tendency to greater average MVIC KE torque **immediately** post-recovery, and **@ 48 h** post-Ex in CWI versus TWI groups	Ø of CWI (versus TWP) on maximal strength **@ 0–1 h and 24–48 h**. ↑ (tendency) of CWI (versus TWI) on maximal strength (average only) @ 0 h and 48 h
Buchheit et al. [113]	Recreationally trained cyclists (10 M, 29 Y)	Cycling: 2 × 1-km TT with 20 min rest. *35 °C with 40% RH*	Crossover design. Immersion after the 1st 1-km TT: CWI: 14 °C for 5 min. CON: 5 min PR	-Similar cycling time and mean cycling PO during the 2nd 1-km TT in CWI and CON	Ø of CWI on subsequent (**@ 20 min**) cycling sprint performance
Crampton et al. [119]	Recreationally trained subjects (8 M, 25 Y)	Cycling: 3 × 30 s all-out cycling bouts with 4 min rest (Ex1). 35 min recovery. 3 × 30 s all-out cycling bouts with 4 min rest (Ex2). *21 °C*	Crossover design. Immersion after Ex1: CWT1/1: 2.5 min at 8 °C and 2.5 min at 40 °C for 30 min. CWT1/4: 1 min at 8 °C and 4 min at 40 °C for 30 min. CON: 30 min PR	Greater peak PO of Ex2 in CWT1/4 versus PR, with no differences between CWT1/1 and CON. Reduced total work during Ex2 in CON but not CWT conditions	↑ of CWT on subsequent (**@ 35 min**) cycling sprint performance
Crampton et al. [114]	Well trained cyclists (8 M, 25 Y)	Cycling: 3 × 30 s all-out cycling bouts with 4 min rest (Ex1). 40 min recovery. 3 × 30 s all-out cycling bouts with 4 min rest (Ex2)	Crossover design. Immersion after Ex1: CWI: 15 °C for 30 min. CON: active recovery (arm Ex) for 30 min. CWI (15 °C) with active recovery (arm Ex) for 30 min. TWI (34 °C) with active recovery (arm Ex) for 30 min	Lower mean PO of Ex2 in CWI versus the other trials. Mean PO of Ex2 only preserved in arm Ex and TWI with arm Ex. Lower peak PO of the 1st sprint of Ex2 in CWI and CWI with active recovery versus the two other trials. Peak PO of Ex2 only preserved in TWI with active recovery	↓ of CWI on subsequent (**@ 40 min**) cycling sprint performance
Crowe et al. [115]	Recreationally trained subjects (13 M, 4 F, 21 Y)	Cycling: 2 × 30 s all-out cycling bouts with 60 min rest	Crossover design. Immersion 10 min after the 1st sprint: CWI: 13–14 °C for 15 min. CON: 15 min PR	Reduced peak PO and total work during the 2nd 30-s all-out cycling sprint in CWI but not CON conditions	↓ of CWI on subsequent (**@ 60 min**) cycling sprint performance
Hurrie and Giesbrecht. [116]	Well trained subjects (6 M, 3 F, 32 Y)	Cycling: 3 × 30 s all-out cycling bouts with 4 min rest (Ex1). 40 min recovery. 3 × 30 s all-out cycling bouts with 4 min rest (Ex2). *20–22 °C*	Crossover design. Immersion after Ex1: CWI (15 °C) with active recovery (cycling) for 30 min. TWI (34 °C) with active recovery (cycling) for 30 min. TWI (34 °C) for 30 min	Reduced mean and peak PO during Ex2 versus Ex1 only observed in CWI with active recovery	↓ of CWI with active recovery on subsequent (**@ 40 min**) cycling sprint performance
Kim and Hurr. [21]	Recreationally trained subjects (11 M)	Cycling: 2 × 30 s all-out cycling bouts with 10 min rest	Crossover design. Recovery intervention after the 1st sprint: Leg cooling suit for 10 min. CON: 10 min PR	Lower peak PO during the 2nd 30-s cycling sprint in cooling versus CON. Lower mean frequency (EMG) during the 1st 10 s of the 2nd sprint in cooling versus CON	↓ of leg cooling on subsequent (**@ 10 min**) cycling sprint performance
Parouty et al. [122]	Well trained swimmers (5 M, 5 F, 19 Y)	Swimming: 2 × 100 m sprints with 30 min rest	Crossover design. Immersion 5 min after the 1st sprint: CWI: 14–15 °C for 5 min (up to the neck). CON: 5 min PR	Higher swimming time during the 2nd 100 m sprint in CWI versus CON	↓ of CWI on subsequent (**@ 30 min**) swimming performance
Schnieep et al. [117]	Well trained cyclists (10 M, 30 Y)	Cycling: 2 × 30 s all-out cycling bouts with 15 min rest	Crossover design. Immersion immediately after the 1st sprint: CWI: 12 °C for 15 min. CON: 15 min PR	Greater decline in mean and peak PO for CWI versus CON during the 2nd 30-s all-out cycling sprint	↓ of CWI on subsequent (**@15 min**) cycling sprint performance
White et al. [121]	Recreationally trained subjects (8 M, 24 Y)	Running: 12 × 120 m maximal sprints with 2.5 min rest	Cross over design: CWI: 10 °C for 10 or 30 min. CWI: 20 °C for 10 or 30 min. CON: 45 min PR	Similar recovery of SJ performance in CWI conditions and CON **@ 1, 2, 24 and 48 h** post-Ex. Similar reduction of DJ performance **@ 1–2 h** post-Ex in all conditions, and full recovery of DJ performance only in 10 °C-CWI (10 and 30 min).**@ 24–48 h** post-Ex	Ø of CWI on SJ performance **@ 1 h to 48 h**. ↑ of CWI (10 °C) on DJ performance **@ 24–48 h**
Yoshimura et al. [118]	Well trained subjects (15 M, 19 Y)	Cycling: 2x (10 min at 50%VO_2max_ + 30 s all-out) with 20 min rest. *25 °C and 50% RH*	Cross over design: CWI: 20 °C for 20 min (up to the neck). CON: 20 min PR. *exclusion of one condition (CWI with CO_2_)	Similar decline in mean PO in CWI and CON during the 2nd 30-s all-out cycling sprint	Ø of CWI on subsequent (**@30 min**) cycling sprint performance
*Repeated post-exercise exposures*					
Broatch et al. [17]	Recreationally trained subjects (16 M, 25 Y)	6-wk cycling training (3 sessions/wk): 4–6 × 30 s all-out bouts with 4 min rest	Immersion after each session, 2 groups: CWI: 10 °C for 15 min. CON: PR	Similar improvement of 2 km-TT and 20 km-TT performance, peak PO and VO2peak (incremental test) in CWI and CON groups	Ø of CWI on endurance performance

Water immersion was applied up to the waist/lower part of the trunk, unless stated otherwise. Text highlighted in italic describes the ambient condition, when stated (air temperature and relative humidity)

Text in bold describes the specific time points

*CMJ* countermovement jump, *CON* control, *CWI* cold water immersion, *DJ* drop jump, *Ex* exercise, *F* female, *KE* knee extension, *KF* knee flexion, *M* male, *MVIC* maximal voluntary isometric contraction, *PO* power output, *PR* passive recovery, *RH* relative humidity, *SJ* squat jump, *TT* time trial, *TWI* thermoneutral water immersion, *TWP* thermoneutral water immersion with placebo, *VO*_*2*_ oxygen uptake, *wk* week, *Y* year, ↑ positive effect, ↓ negative effect, Ø no effect

*One condition was excluded because it was not relevant for the purpose of the review

Various modes of cooling and heating have been described in a recent review [3]. Here, we included studies employing post-exercise CWI of the exercised limbs or whole-body CWI (immersion up to the neck or including the head). The majority of these studies used immersion into 5–20 °C water for 5–30 min (see Tables 1, 2, 3). We also included some studies employing ice-chilled cuffs and other body cooling systems [19–21]. The few studies employing post-exercise heating used hot water immersion (HWI) of the lower limbs or whole-body HWI (38–40 °C for 15–40 min) [16, 22–24], hot water-perfused arm-cuff [19] or heat pad [25] (see Tables 1, 2, 3). Contrast water therapy (CWT), which consists of alternating CWI and HWI for 10–30 min, is also commonly used during recovery, in particular after endurance exercise (see Table 2). Studies included in this review only employed water immersion or methods that cover the exercised limbs (cuffs and pads). A cryotherapy chamber cools the air while conversely sauna heats it, and similarly to water, air is a medium that cools/heats the body by convection and conduction. However, water is > 800 times denser than air [26], and the thermal conductivity by water is 25 times greater than by air [27]. In addition, cryochamber and sauna are used in different manners than the other methods presented above (e.g., exposure to extremely low and very high temperatures, respectively, application during only short durations with a cryochamber). For these reasons, studies using cryotherapy chambers or sauna were not included in this review. Passive recovery (sitting at room temperature) was generally used as a control condition, while other alternative control recovery methods were sometimes proposed, such as thermoneutral water immersion (TWI) and active recovery. Water temperature of ~ 35 °C is usually considered as thermoneutral because it does not change core temperature during prolonged immersion. Lower water temperatures (28–34 °C) were also considered as thermoneutral as they unlikely to cause reduced body temperature during moderate exposure [28]. It is important to highlight that most studies included in this review employed young healthy male subjects who were recreationally active or well-trained. Therefore, the described cooling/heating effects (or absence of effect) should be interpreted with caution when other populations are considered.

## Resistance Exercise

Numerous studies which evaluated the effects of cooling or heating have assessed maximal voluntary isometric contraction (MVIC) torque [20, 25, 29–33], 1 repetition maximum (1-RM) strength [34, 35], or electrically evoked muscle force [20], as primary functional outcome measures (Table 1). Other studies have employed various jump tests to assess the post-exercise recovery of power generation which is relevant to fast movements in sports [29]. Several studies have also determined muscle fatigue resistance measured as the total work performed over a resistance exercise session [29, 30, 32, 34, 36–39].

### Early Recovery Phase (5 min to 6 h)

Four studies have examined the effects of CWI on the recovery of neuromuscular function in the immediate post-recovery period up to 6 h following resistance exercise [20, 29, 30, 33]. In the immediate (5–40 min) post-recovery period, knee extension MVIC torque was recovered following 10 min CWI at 10 °C, whereas it remained lower than pre-exercise values in the control condition (active recovery) [30]. At the 2 h post-exercise recovery period, Pointon and colleagues showed that the recovery of knee extension MVIC torque and voluntary activation were similar between the control rested condition and the cooling condition (ice cuff covering the entire exercised leg for 20 min immediately post-exercise) [20]. Another study by Roberts et al. (2014) assessed the recovery of neuromuscular function in the 2–6 h post-exercise recovery period [29]. Maximal muscle function determined by jump performance or peak isometric strength was not different in the CWI versus control conditions. However, fatigue resistance determined as average and total load lifted during 6 sets × 10 squats at 80% 1-RM was increased in the CWI versus control conditions at 6 h post-exercise. Conversely, a subsequent paper by Roberts and colleagues showed that fatigue resistance determined as total work performed during 50 repetitions of single legged isokinetic knee extensions at 90°/s was similar in the CWI versus control conditions at 1 h post-exercise [30]. Similarly, no benefits of CWI were evident for the recovery of MVIC and jump performance within 4.25 h after a resistance exercise session [33].

One proposed mechanism by which CWI may increase the recovery of strength during the 5–40 min post-recovery period is by reducing pain perception through inhibiting the metabolite sensitive III and IV muscle afferents [40], which are sensitive to increases in [lactate], [H^+^], and high [ATP] [41, 42]. Although the accumulation of pain-inducing metabolites such as lactate is relatively high during resistance exercise including multiple sets performed at maximal intensity [43, 44], the majority of lactate removal occurs within 15 min post-exercise [45], arguing that this mechanism of pain inhibition will only explain the short-term enhancement of muscle function recovery mediated by CWI. Another hypothesis is that the acute effects of CWI on muscle function relate directly to temperature-dependent effects on excitation–contraction coupling processes. Indeed, it has been shown in the non-fatigued state that local muscle cooling induces a leftward shift in the force-frequency relationship with the force enhancement most evident at low compared with high activation frequencies [46, 47], which is likely caused by both a slowing of sarcoendoplasmic reticulum calcium transport ATPase (SERCA) pumps and cross-bridge kinetics augmenting pulse-induced force fusion [47, 48]. However, the temperature reduction following 10 min CWI at 10 °C in the vastus lateralis muscle (1 cm depth) is variable ranging from only 1–2 °C to as much as 12 °C among subjects [29], suggesting a modest role of temperature on altered excitation–contraction coupling. Therefore, any benefits of acute cooling on enhanced muscle force generation appear to be modest.

### Later Recovery Phase (24–72 h)

In the post-exercise recovery period lasting 24–72 h, the effects of immediate post-exercise muscle cooling on muscle function are likely to have disappeared as the muscle recovers to its physiological temperature. For instance, Wilson et al. (2019) showed that following a lower body resistance exercise session, the recovery of knee extension MVIC torque, maximal isometric squat strength, and 60°/s maximal isokinetic knee extension torque 24–72 h following 10 min CWI at 10 °C were similar or worse than in the control placebo condition [31]. In addition, Pointon et al. (2011), who performed 20 min cooling using an ice cuff following single leg isokinetic knee extensions, showed no differences in sarcolemmal membrane excitability (measured by M-wave amplitudes from surface EMG), submaximal torque (potentiated twitch torque) or MVIC torque 24–48 h post-exercise, compared with the control condition [20]. These two studies suggest that muscle contractile function is either impaired or not affected by CWI when assessed at 24–72 h following resistance exercise.

Fatigue resistance has also been assessed in the 24–48 h period following a single session of resistance exercise and subsequent 10 min CWI at 10–12 °C [36, 37]. Fatigue resistance during resistance exercise is primarily dictated by intramuscular factors [49]. Muscle metabolite accumulation during this high-intensity type of brief duration exercise leads to rapid force loss from increased inorganic phosphate (P_i_) and hydrogen ion (H^+^) affecting predominately sarcoplasmic reticulum (SR) Ca^2+^ release, myofibrillar Ca^2+^ sensitivity, and cross-bridge force generation [12, 13, 50]. However, clearance of metabolites largely occurs within 5–10 min following fatigue induction [51, 52], arguing that any effect of acute CWI on metabolite clearance will have no consequence on fatigue resistance during a subsequent exercise test performed 24–48 h later. Indeed, in the two studies that previously assessed fatigue resistance at 24–48 h following a single resistance exercise session, no difference in fatigue resistance was determined between the CWI versus control groups based on the total number of squat repetitions at 80% 1-RM, and average squat power per repetition over four sets of exercise [36, 37]. In summary, CWI following resistance exercise does not affect fatigue resistance after 24–48 h.

### Repeated Post-exercise Cooling/Heating (4–12 Weeks of Training)

Several studies have investigated whether repeated use of CWI during resistance training intervention affects muscle strength improvements (Table 1). In the shorter 4–8-week training period, six studies performed resistance exercise involving wrist flexion or handgrip [32, 38, 39], knee flexion [34], or a combination of leg exercises [53] or upper and lower body exercises [35]. These exercises were followed immediately by limb [32, 34, 38, 39] or whole body CWI [35, 53] lasting 12–20 min in 10–14 °C water. Compared with the control condition, CWI generally showed no effect in altering maximal strength or power gains across these studies, except in one study [38]. No improvement of maximal strength was observed after training in some of the aforementioned studies for both the CWI and control groups/conditions [39, 53], which may have erased potential negative effects of cooling on force development. Neural adaptation is the main factor responsible for the gains in strength in the first 8 weeks of resistance training [54]. Although it remains to be investigated, neural adaptation to resistance training may not be affected by CWI, which could explain the absence of any effect of CWI on strength development after a short training intervention.

When the training period was extended to 12 weeks, post-exercise CWI (10 min at 10 °C) blunted the increases in MVIC torque compared with the control condition (active recovery) [55]. In the same study, the CWI-induced impairment in muscle strength gains appeared consistent with the findings of reduced muscle hypertrophy, especially in type II muscle fibers, and reduced activation of anabolic signaling. CWI (15 °C for 15 min) blunted testosterone response after a bout of resistance exercise [56] and CWI (8 °C for 20 min) applied after every resistance exercise session decreased daily muscle protein synthesis rates over a 2-week training period [57]. These findings are in line with chronic impairments in the muscle hypertrophic response observed previously [55]. While muscle protein turnover is dictated by both synthesis and breakdown processes, CWI does not seem to affect muscle protein breakdown [35].

With regard to fatigue resistance and the use of CWI over a 4–12-week resistance training period [32, 34, 38, 39, 55], the results have consistently shown a blunted muscle fatigue resistance with CWI compared with the control condition. Muscle fatigue resistance adaptations following high-intensity exercise could be triggered by reactive oxygen and nitrogen species (RONS) induced adaptations [58, 59], whereby abolishing the exercise-induced increase in muscle RONS generation with antioxidant supplementation blunted long-term endurance training adaptations [60–64]. RONS generation remains elevated in the recovery period after exercise [65] and CWI employed in the immediate post-exercise recovery period may diminish RONS production [66]. Thus, it could be speculated that cooling-induced reduction in RONS generation during the recovery phase might blunt fatigue resistance adaptation during resistance training. However, as presented in the next section, there is no clear evidence that post-exercise CWI impairs the development of endurance performance, suggesting that RONS-independent mechanisms are responsible for CWI-mediated reduction of fatigue resistance adaptation.

Given the detrimental effect of CWI on resistance training adaptations, a natural progression has been the investigation of HWI as a recovery modality on strength adaptations following resistance training. In a recent 12-week resistance training study, a heat pad (~ 40 °C) was wrapped around one thigh during the knee extension exercise session as well as during the 20-min post-exercise recovery period of every resistance training session [25]. These authors showed that the training-induced increases in knee extension isokinetic torque (90°/s) were not affected by heating compared to the contralateral untreated leg. This is consistent with the absence of effect of HWI (20 min at 46 °C) on muscle protein synthesis rates after a resistance exercise session, compared with control condition [67]. The above studies suggest that short-term muscle heating during/after resistance training sessions might not provide an additive stimulus for improving long-term muscle strength adaptations. Compared with CWI which has a long-lasting muscle cooling effect that can persist for a few hours after immersion [68, 69], muscle temperature returns to pre-exercise values within 30 min after HWI [67]. Different results might be observed when prolonged periods of post-exercise HWI are applied during a resistance training intervention, due to the extended time of high muscle temperature after exercise. Indeed, some human studies indicate that prolonged passive heating applied on the thigh could induce a slight skeletal muscle hypertrophy (3–6% increase in muscle cross-sectional area) and gain in muscle strength (~ 6% increase in MVIC of knee extensors) without exercise training (heat-and-steam-generating sheet applied 8 h/day, four times/week for 10 weeks) [70] or could limit skeletal muscle atrophy associated with immobilization (2 h daily treatment with pulsed shortwave diathermy for 10 days) [71]. A recent study also found that 90-min passive heating repeated over 8 weeks (unilateral thigh heating with ~ 52 °C water-circulating garments, 5 days/week) slightly increased maximal isokinetic knee extension torque (5%) [72]. Future studies should investigate whether prolonged post-exercise muscle heating is effective in improving resistance training-associated adaptations.

## Summary

- Early recovery phase (5 min to 6 h): possible increase in maximal strength recovery following CWI between 5–40 min post-exercise, but not at later recovery timepoints; possible positive effect of CWI on fatigue resistance (1 h post-recovery to 6 h post-exercise).
- Later recovery phase (24–72 h): no effect or negative effect of CWI on muscle strength recovery (24–48 h); no effect of CWI on fatigue resistance (24–48 h).
- Post-exercise recovery on chronic resistance training adaptations (4–12 weeks): no effect (4–8 weeks) or negative effect (12 weeks) of CWI on muscle strength gains; no effect of local heating on muscle strength gains; negative effect of CWI on muscle fatigue resistance.

## Endurance Exercise

Endurance exercise results in numerous physiological perturbations including metabolite accumulation, hyperthermia, dehydration and glycogen depletion, which ultimately lead to fatigue and reduced performance [73]. In addition, several physiological changes are observed following endurance training intervention, such as cardiovascular and skeletal muscle adaptations [73]. Numerous studies investigated the effect of post-exercise cooling, heating, or CWT on the recovery of endurance performance, while other studies employed various neuromuscular tests to assess the recovery of muscle strength (e.g., MVIC, isokinetic force) and explosiveness (jump and sprint performances). Finally, application of post-exercise cooling or heating during regular endurance training was proposed to influence physiological adaptations and endurance performance. A summary of these studies is presented in Table 2.

### Early Recovery Phase (Immediate to 9.5 h)

Post-exercise CWI, compared with control condition, could enhance recovery of endurance performance and fatigue resistance in the immediate period (0 to 40 min) following recovery intervention [74–81]. Crampton et al. (2013) observed that the time to exhaustion during cycling (80% VO_2max_) starting 5 min after recovery intervention was longer in the CWI (15 °C for 30 min) than the other recovery conditions (active recovery, CWT and TWI) [75]. A longer time to exhaustion was also observed during intense running (~ 90% VO_2max_) in the CWI condition (8 °C for 15 min) than passive recovery, while no differences were found with warmer water (15 °C for 15 min) [76]. This positive effect of CWI was generally observed when exercise was performed in thermoneutral (~ 20 °C) [77] or in hot environments [78–80]. In contrast, CWI (15 °C for 15 min) performed after a strenuous endurance session in the heat (30 °C and 50% relative humidity) did not significantly improve subsequent endurance performance (lowest cycling time to perform a work equivalent to 12 min at 85% Pmax) compared with passive recovery, when assessed 45 min after recovery intervention [82]. Core temperature increases during prolonged and intense lower limb endurance exercise executed in thermoneutral conditions, and this increase is accentuated in hot conditions [83]. CWI can accelerate the post-exercise reduction in core temperature, allowing maintenance of a cooler core body temperature during the subsequent endurance session [78–80, 82]. Thus, CWI could improve the heat storage capacity and reduce the thermoregulatory demand for heat dissipation, thereby alleviating cardiovascular strain, enhancing blood flow redistribution to active muscle, reducing perceived effort, and limiting central fatigue and dehydration [6].

In contrast, post-exercise CWI (14–15 °C for 5–15 min) does not improve the performance of a subsequent endurance exercise performed in thermoneutral conditions when the period between the two sessions is more prolonged (3 to 9.5 h) [22, 84, 85], despite an improved perceived recovery [84]. Rectal temperature measured during a 5-km running time trial performed ~ 4 h after an initial exhaustive endurance exercise was identical between CWI and passive recovery trials [22], indicating that thermal stress during the second session was certainly similar in both conditions. To conclude, although numerous factors need to be considered (e.g., duration and intensity of exercise, ambient conditions, CWI protocol, etc.), post-exercise CWI only improves subsequent endurance performance when the period between the two sessions is short (< 1 h), and this is possibly due to the lowered heat stress applied on the body during the second exercise bout. In addition, it remains to be investigated whether the beneficial effect of CWI on subsequent endurance performance in the heat persists for longer periods (i.e., 3–6 h).

We have recently shown in recreationally active subjects that prolonged local post-exercise cooling (2 h with ice-chilled water-perfused arms) negatively affects fatigue resistance during a subsequent high-intensity arm-cycling session (3 × 5 min all-out bouts) performed ~ 10 min after cooling [19]. Core temperature was ~ 36.5 °C during the 2 h-recovery period and was not reduced by local cooling, suggesting that cooling did not change thermal stress during subsequent exercise. In this study, cooling induced a severe drop of the temperature in triceps brachii muscle during the 2-h-recovery period (~ 17 °C on average at 1.5 cm depth), while the drop in vastus lateralis muscle temperature after conventional CWI protocols is generally much lower (~ 3–5 °C on average at 1–3 cm depth) [29, 69, 86]. Although not investigated by Cheng et al. [19], severe local cooling may have induced arterial vasoconstriction in the upper limbs, thereby potentially reducing muscle blood flow, oxygen and nutrient delivery, and metabolite clearance during subsequent exercise.

In contrast, prolonged local heating (38 °C heated arm cuff for 2 h) improved fatigue resistance during 3 × 5 min all-out arm-cycling bouts performed in thermoneutral conditions ~ 10 min after heating [19]. Another study found that short HWI (38 °C for 15 min) did not affect 5-km running performance (compared with passive recovery) in thermoneutral conditions ~ 4 h following the initial bout [22]. These controversial findings could result from the duration and form of local heating, and from the exercise test performed. Although it remains to be investigated, prolonged post-exercise heating may impair subsequent endurance performance executed in hot ambient conditions due to the reduced heat storage capacity and increased demand for heat dissipation, which consequently would exacerbate thermal and cardiovascular strains.

Four studies investigated the impact of CWT executed after a strenuous endurance session on the subsequent performance [75, 84, 87, 88]. Three of these four studies observed a beneficial effect of post-exercise CWT on subsequent endurance performance executed up to 2 h after the initial exercise session [75, 87, 88], while no effect was found when the period between the two sessions was more prolonged (3 h 15 min) [84]. In addition, short exposure to CWT (6 min) was more beneficial than longer exposure (18 min), at least when subsequent exercise is performed 2 h following the first session [87, 88]. Finally, CWT (8 °C/40 °C for 30 min) was not as effective as CWI (15 °C for 15 min) in improving the time to failure at 80% VO_2max_ when exercise was performed 5 min post-recovery [75].

Ex vivo experiments showed that exposing mouse intact single muscle fibers to 26 °C for 1 h directly after a fatiguing protocol impaired subsequent endurance performance (repeated tetanic contractions) compared with 36 °C, confirming the temperature-dependent effect on fatigue resistance [19]. This finding was associated with a slower glycogen repletion at 26 °C than 36 °C in whole muscle. Similarly, 4-h exposure to local heating (heat pack) promoted glycogen resynthesis in the human vastus lateralis muscle after cycling [89], while the opposite result was observed after a 4-h intermittent exposure to ice cooling (plastic bags) [90]. In contrast, short exposure to CWI (10 min at 8 °C) did not impair muscle glycogen repletion [86]. Prolonged local heating may influence muscle glycogen repletion by modulating glucose delivery and muscle glucose uptake, and by affecting the activity of glycogen synthase and phosphorylase [91–93]. Altogether, prolonged local cooling or heating seems to influence muscle glycogen resynthesis following exhausting endurance exercise. This could have a direct impact on endurance exercise capacity when the subsequent exhausting endurance session is highly dependent on muscle glycogen as an energy substrate, and the exercise is performed within a few hours (4–8 h) following the first session.

There are limited data available on the effect of post-endurance exercise cooling on early recovery of neuromuscular function. Countermovement jump (CMJ) performance and muscle strength (MVIC of knee extensors) were impaired during the immediate period following post-exercise CWI (14–15 °C for 15–20 min vs. passive recovery) [94, 95]. However, no effect of CWI was observed on MVIC [96], isokinetic force [96, 97] and jump performance [97] in the immediate phase following recovery intervention when the initial endurance exercise was performed in the heat (30–40 °C). At the single fiber level in mice, 0.5–2 h cooling was shown to impair the recovery of contractile force after low-intensity fatiguing stimulation, especially at submaximal levels of activation, and this was attributed to impaired SR Ca^2+^ release [19]. A limited number of ex vivo studies from mouse skeletal muscle showed a beneficial effect of heating on submaximal force recovery, which was associated with enhanced muscle glycogen re-synthesis and faster restoration of SR Ca^2+^ release [19, 91, 93]. To date, the effect of heating on early recovery of human neuromuscular function remains to be investigated.

### Later Recovery Phase (24–72 h)

Post-exercise CWI (10–12 °C for 5–10 min) did not improve 5-km running performance when the between-session recovery period was 24 h [98]. However, running performance (number of shuttles completed during a Yo-Yo intermittent recovery test) performed 24 h after interval exercise (8 × 3 min) was slightly improved when the initial exercise session was followed by CWI (15 °C for 15 min) compared with passive recovery [74]. The effect of post-exercise local heating on endurance performance during the later recovery phase remains to be investigated. As discussed in "Early Recovery Phase (Immediate to 9.5 h)" section, prolonged local heating and cooling following exhausting endurance exercise seems to influence muscle glycogen repletion, and this may have a direct impact on the subsequent performance when endurance exercise is performed 4–8 h after the initial session. Since muscle glycogen stores are usually fully replenished within 24–48 h after prolonged endurance exercise when carbohydrate is provided adequately [99], local cooling or heating following glycogen-depleting exercise would unlikely affect performance of endurance exercise bouts performed 1–2 days after the initial bout.

CWI (8–15 °C for 10–15 min) did not influence the later post-exercise recovery of muscle strength (peak isokinetic torque and MVIC, 24–48 h post-exercise) and jump performance [drop jump (DJ), 24–48 h post-exercise; CMJ, 24–48 h post-exercise; triple hop, 24 h post-exercise] [94, 97, 100, 101]. In these studies, these outcomes were only slightly impaired directly after the initial endurance session and were fully recovered within 24–48 h. Future experiments could focus on the impact of post-exercise cooling in extreme endurance activities (such as mountain ultra-marathon) where neuromuscular function is substantially impaired directly after exercise [102, 103] and required several days to be fully recovered [103].

### Repeated Post-exercise Cooling/Heating (up to 5 Weeks of Training)

Post-exercise CWI during an aerobic training program (4 weeks) did not promote or limit the development of endurance performance [32, 104]. Moreover, post-exercise CWI during an intense 39-day cycling training intervention (1–2 sessions/day) did not affect 10-min time trial performance, but magnitude-based inferences showed that CWI had an unclear greater increase in 2 × 4-min mean power and a likely greater increase in mean sprint power compared with passive recovery [105]. As described in recent reviews [3, 4], local cooling (CWI mostly studied) after endurance exercise is able to stimulate some molecular actors regulating muscle adaptation and angiogenesis. For instance, CWI was shown to potentiate the exercise-mediated upregulation of peroxisome proliferator-activated receptor gamma coactivator 1-alpha (*PGC1A*), a major regulator of mitochondrial biogenesis [69, 106, 107]. Post-exercise CWI also increased the mRNA levels of vascular endothelial growth factor (*VEGF*), a gene promoting angiogenesis [69], a result associated with an enhanced microvascular adaptation [108]. Currently, there is no clear evidence that post-exercise CWI does potentiate the gain in endurance performance. Future research including longer training interventions would allow to draw clear conclusions about the role of CWI in augmenting endurance training adaptations.

To our knowledge, only one study investigated the influence of post-exercise heating on the development of endurance performance during a training intervention [23]. Six elite short-track speed skaters trained over a 4-week period (18 h/week) and HWI (40 °C for 20 min) was applied after the last session of the day. In general, HWI did not affect endurance and sprint performance compared with passive recovery. However, it had a positive effect on maximal strength (MVIC) and the tendency to increased VO_2max_ (*P* = 0.053) in response to the training intervention was only found in the HWI condition. Except for small improvement (effect size *d* ~ 0.2) of half-lap time and total time during the 1.5-lap all-out ice-skating exercise in the passive recovery condition, this short training program did not significantly improve the performance of the other field tests on ice (3-lap and 7-lap all-out exercises) in these elite athletes. In addition to the small sample size, the absence of improvement or small improvements of exercise capacity after training intervention may have minimized the chance of detection of potential HWI-mediated benefits on enhancing sport performance in these elite short-track speed skaters.

A recent study on sedentary subjects observed that compared to the control unheated leg, 2-h local heating (pulsed shortwave diathermy) increased the phosphorylation of AMP kinase (AMPK) and extracellular signal-regulated kinase 1/2 (ERK1/2), both associated with mitochondrial biogenesis [109]. The same study showed that repeated exposures to the same heating method (2 h daily for 6 consecutive days) increased PGC1A protein levels and maximal mitochondrial respiratory capacity [109]. In contrast, 1 h local heating of one leg (water-circulating sleeve connected to a bath circulator set at 49.5 °C) did not affect the expression of any markers of mitochondrial biogenesis in recreationally trained individuals [110]. In addition, acute local heating (lower body heating or unilateral thigh heating with 48–52 °C water-circulating garments for 90 min) increased the mRNA levels of factors associated with capillary growth [e.g., *VEGF* and angiopoietin 2 (*ANGPT2*)] in skeletal muscle of recreationally active subjects [111]. Repeated exposures to local heating for 8 weeks (unilateral thigh heating with ~ 52 °C water-circulating garments for 90 min, 5 days/week) increased the protein content of endothelial nitric oxide synthase (eNOS) and had a positive effect on the capillarization of type-II muscle fibers compared with the control condition, while it did not affect mitochondrial content [72]. Whether prolonged post-exercise local heating could promote the development of muscle oxidative capacities, capillarization and endurance adaptations over a training intervention (> 8 weeks) remains to be investigated.

To date, two studies investigated the impact of repeated uses of post-exercise HWI, CWI or CWT during a short intervention period (5–6 days) on the maintenance of physical performance [16, 24]. Daily exposure (immersion up to the neck) to CWI (15 °C for 14 min) or CWT (15 °C/38 °C for 14 min) following prolonged cycling was effective in maintaining sprint cycling performance, while HWI (38 °C for 14 min) had no effect [16]. Zurawlew et al. showed that daily HWI (40 °C for 40 min) following 40 min running (65% VO_2max_) improved 5-km running performance (compared to TWI at 34 °C for 40 min) in hot (33 °C) but not temperate (18 °C) environments [24], indicating that HWI could be relevant for improving acclimation to heat.

### Summary

- Early recovery phase (immediate to 9.5 h): CWI (≤ 30 min) improves only endurance performance when the second exercise is performed < 1 h after the initial session. One study indicates that prolonged local cooling (2 h) impairs endurance performance recovery immediately after cooling, while prolonged local heating (2 h) is beneficial. There is no or negative effect of CWI on jump performance and maximal strength immediately after recovery intervention.
- Later recovery phase (24 to 72 h): no effect of CWI on recovery of neuromuscular function and endurance performance.
- Post-exercise recovery on chronic endurance training adaptations (up to 5 weeks): no clear effect of CWI and HWI on improvement of endurance performance.

## Sprint Exercise

Training including short and prolonged sprints promotes the development of peak power output (PO) and anaerobic capacity, which are required in most competitive sports. Sprint interval training can also elicit skeletal muscle and cardio-respiratory adaptations generally observed following endurance training [73, 112]. Several studies have investigated the impact of cooling [21, 113–118] and CWT [119] following one or 3–4 bouts of all-out sprint exercise on the recovery of peak PO, mean PO or total work during identical sprint exercise performed 15–60 min after the initial session. In addition, a few studies evaluated the effects of cooling applied after repeated sprints on the recovery of maximal strength (MVIC) [120] and jump performance [121]. Finally, the role played by post-exercise cooling during a 6-week sprint training intervention on training adaptations was recently investigated [17]. A summary of these studies is presented in Table 3. To our knowledge, the impact of post-exercise heating following sprint exercise has not yet been investigated.

### Early Recovery Phase (Immediate to 2 h)

The majority of the studies indicated that CWI (12–15 °C for 5–30 min) or local cooling (leg cooling suit) applied after 1–3 bouts of 30-s all-out cycling sprint (Wingate tests) impaired sprint performance (mean and/or peak PO) during a subsequent exercise performed 15–60 min after the initial session [21, 114–117]. In addition, whole-body CWI (14–15 °C for 5 min) performed 5 min after a single swimming sprint (100 m) slightly impaired the performance of a subsequent sprint (compared with passive recovery) executed 30 min after the initial exercise [122]. In contrast to prolonged endurance exercise, 1–3 bouts of all-out cycling sprint performed in thermoneutral conditions induced a limited increase in core temperature (0.2–0.5 °C) [114, 115], which was then reduced to levels below baseline (36.5 vs. 37.3 °C) after the 1st repeated sprint bout following CWI (15 °C for 30 min) [114]. Muscle temperature (4 cm into vastus lateralis muscle) increased from ~ 36 °C to ~ 38 °C after 4 Wingate tests and was then reduced to ~ 33.5 °C directly after CWI (10.3 °C for 15 min) [120]. Reduced muscle and core temperatures following CWI have been proposed to be the major factors responsible for the detrimental effect of cooling on sprint performance [114, 115, 117, 122]. Older research showed that peak PO during cycling sprint declined proportionally to the reduction of muscle temperature (from 38 to 30 °C), a result attributed to a slower rate of force development [123]. In addition, this negative effect on performance is more marked for fast than slow movements [123, 124], which could explain why the detrimental effect of CWI is so apparent during Wingate tests. Muscle cooling could also increase muscle stiffness [125], as well as reduce enzyme activity [126], leading to a reduced anaerobic ATP production during sprint exercise. Furthermore, CWI leads to parasympathetic activation and sympathetic withdrawal [113], which could blunt the heart rate acceleration, thereby limiting cardiac output and O_2_ delivery to exercising muscles during the maximal sprints. O_2_ and nutrient supply during exercise could also be diminished by muscle cooling due to peripheral vasoconstriction and reduced blood flow to active muscles [115, 127]. Finally, cooling-induced impairment in sprint performance could be partly related to a modification of neural drive and firing rate of motor units, and to reduced nerve conduction [124, 128]. Altogether, it appears that both peripheral and central factors contribute to impaired sprint performance after cooling.

Two studies did not observe any negative effect of CWI applied after a single cycling sprint on subsequent sprint performance [113, 118], a finding possibly explained by the absence of reduced temperature during the second sprint bout. In the study from Buchheit et al. (2009), trained cyclists performed a 1-km cycling sprint (~ 80 s) in hot conditions (35 °C) before exposure to CWI (14 °C for 5 min) or passive recovery [113]. Rectal temperature was not different between the 2 conditions after exposure and during the subsequent 1-km cycling sprint. In the study by Yoshimura et al., a Wingate test was performed following 10 min low intensity exercise, and whole-body CWI (20 °C for 20 min) did not reduce sublingual temperature during the 2nd sprint [118]. Another study found that CWT (1 min at 8 °C and 4 min at 40 °C for 30 min: CWI/HWI = 1/4) applied after 3 Wingate tests resulted in an improved maintenance of peak PO compared with passive recovery during subsequent cycling sprints [119]. However, this result was not as obvious when CWT alternated a 2.5-min cold/2.5-min hot treatment (CWI/HWI = 1/1). Altogether, these findings suggest that for a sake of faster recovery, core and muscle temperatures should remain equal or slightly above normothermia following sprint exercises, and an additional slight increase in core/muscle temperature due to external heating may eventually be beneficial for improving subsequent sprint performance.

Only two studies investigated the effects of post-exercise CWI on the recovery of jump performance (SJ and DJ) [121] and muscle strength (MVIC) [120] during the early period (1–2 h) following sprint exercise. White et al. (2014) found that CWI conditions (10–20 °C for 10–30 min) were not effective compared with passive recovery in restoring SJ and DJ performance when assessed at 1 and 2 h following 12 maximal running sprints (120 m) [121]. In the study by Broatch et al. (2014), 4 Wingate tests were performed before application of CWI (10.3 °C for 15 min), TWI (34.7 °C for 15 min) or TWI combined with a placebo (skin cleanser added to the water) [120]. Knee extension MVIC torque was similar directly after recovery intervention and at 1 h after exercise in both CWI and TWI-placebo conditions, while TWI alone had a negative effect on MVIC torque compared with TWI-placebo. To conclude, CWI is not effective in accelerating the recovery of jump performance and muscle strength immediately after recovery and 1–2 h following sprint exercises.

### Later Recovery Phase (24–48 h)

The effects of post-exercise CWI on the recovery of jump performance (SJ and DJ) [121] and muscle strength (MVIC) [120] were assessed during the later recovery phase (24–48 h) following sprint exercises. In contrast to the absence of effect of CWI on SJ performance, DJ performance was fully recovered in CWI condition but not in passive recovery condition at 24 h (10 °C for 10 and 30 min) and 48 h (10 °C for 10 min) following sprint exercises [121]. It was previously speculated that CWI could be inhibiting the type III and IV muscle afferents, which may facilitate the stretch reflex and its subsequent contribution to force production during DJ [121]. Furthermore, Broatch et al. showed that MVIC knee extension torque was greater in TWI (34.7 °C for 15 min) combined with a placebo (skin cleanser added to the water) compared with TWI alone at 24 h (average torque) and 48 h (peak and average torques) after sprint exercises [120]. Peak MVIC torque was not different between CWI (10.3 °C for 15 min) group and the two other groups (TWI and TWI-placebo) at 24 and 48 h post-exercise. Moreover, a tendency to greater average MVIC torque (*P* = 0.08) was shown at 48 h after exercise in the CWI group compared with the TWI group in this study, suggesting that the benefits of CWI on recovery of muscle function are partly placebo related [120]. Altogether, the findings of these two studies suggest that CWI applied after repeated sprint exercises has some benefits in accelerating the restoration of neuromuscular function during the later recovery phase.

### Repeated Post-exercise Cooling

To our knowledge, only one study assessed the effects of repeated use of CWI on training adaptations following an intervention including sprint exercises [17], where recreationally active subjects performed 4–6 × 30 s all-out cycling sprints with 4 min of rest 3 times per week for 6 weeks. Performance (2 km and 20 km cycling time trials), VO_2peak_ and peak PO during incremental testing improved similarly with training for the group exposed to CWI (10 °C for 15 min after each session) and the control group (passive recovery). In the same study, CWI did not affect the acute and long-term molecular responses associated with muscle mitochondrial biogenesis and oxidative metabolism. Although it is difficult to draw definitive conclusions from one study, CWI employed after sprint exercises does not appear effective for augmenting endurance-training adaptations. Whether it would affect the development of sprint performance remains to be investigated.

### Summary

- Early recovery period (immediate to 2 h): negative effect of CWI and local cooling on sprint performance; no effect on recovery of jump performance and muscle strength.
- Later recovery period (24–48 h): some benefits of CWI on jump performance (DJ only) and muscle strength.
- Post-exercise recovery period on training adaptations: no effect of CWI.

## Conclusions

**Fig. 1:**
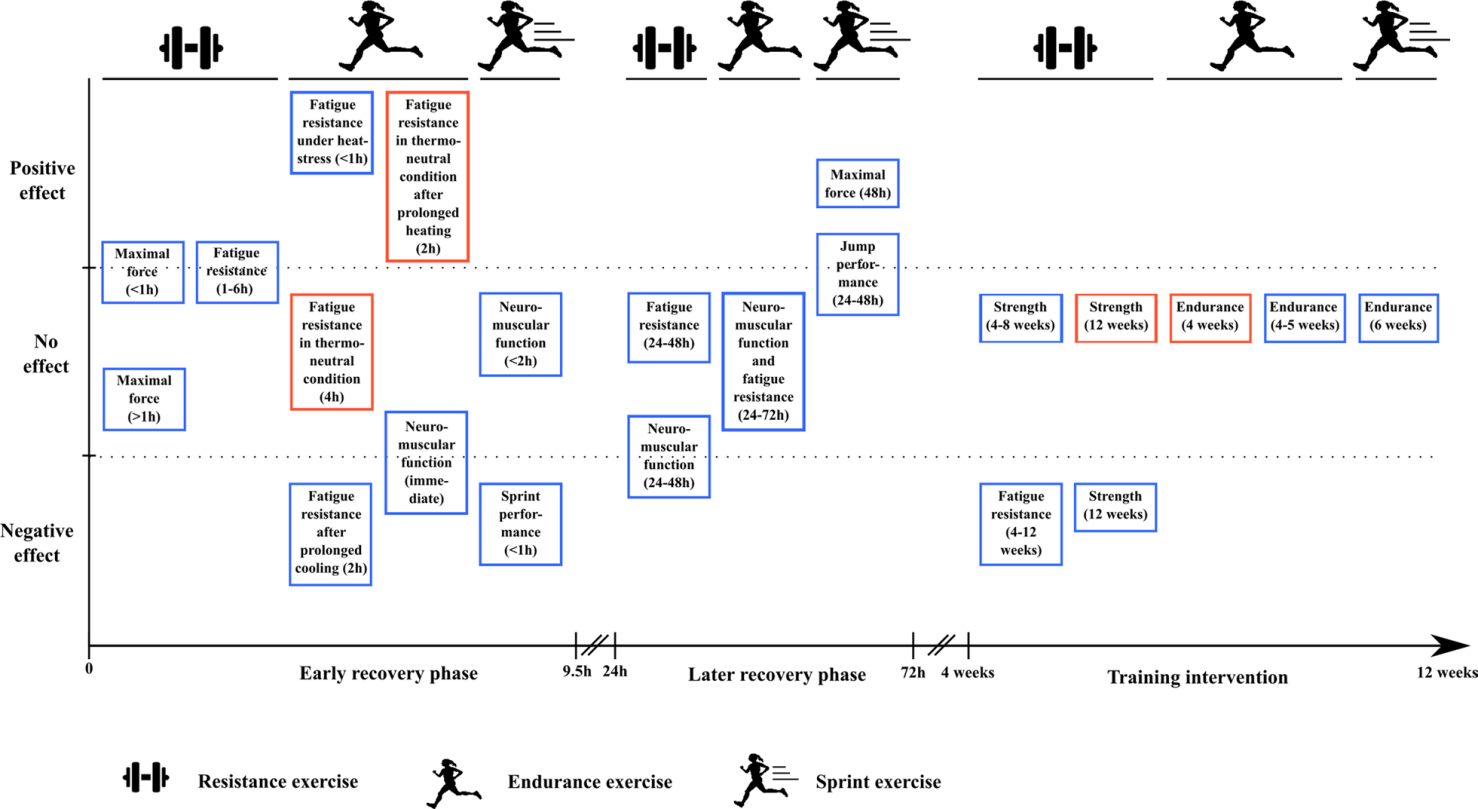
Main effects of post-exercise cooling and heating on functional recovery and training adaptations. The blue boxes illustrate the specific effects of cooling, and the red boxes illustrate the specific effects of heating. These effects are based on the main conclusions drawn from the literature research and from Tables 1, 2, and 3

This comprehensive review analyzed the effects of post-exercise cooling and post-exercise heating on functional recovery and training adaptations and provides practical implications for coaches and athletes wanting to optimize recovery and performance in their sport. A summary of the main effects of post-exercise cooling and heating is presented at the end of each section and is illustrated in Fig. 1. The effects of post-exercise cooling or heating depend on (1) the form and intensity of exercise performed, (2) the functional outcomes being assessed, (3) the post-exercise time period studied, (4) the ambient conditions, (5) the recovery protocol used and (6) whether recovery methods are used acutely or repeatedly. Other factors not discussed in this review may influence the effects of post-exercise cooling or heating such as the training status, sex [129], body composition [130], the genetic background [131], and the previous chronic exposure to cooling or heating.

We identified limited data employing heating as a recovery intervention in the various forms of exercise, and further research is warranted in this area, with the most promise being the use of HWI as a recovery intervention following endurance and sprint exercises. Major limitations of studies employing cooling and heating include the lack of a consistent control condition (e.g., active recovery, passive stretching, sitting), a lack of standardized cooling and heating protocols, the limited data available in elite athletes, and the paucity of long training interventions employing cooling/heating recovery methods. Moreover, since the benefits of CWI appear to be partly placebo related [120], it is surprising that only three studies included in this review used placebo methods [31, 100, 120]. A strong effort should be made in future studies to develop effective placebo conditions.

## Data Availability

Not applicable.

## Abbreviations

1-RM: 1 Repetition maximum
AMPK: AMP kinase
ANGPT2: Angiopoietin 2
CMJ: Countermovement jump
CON: Control
CONC: Concentric
CWC: Cold water cuff
CWI: Cold water immersion
CWT: Contrast water therapy
DJ: Drop jump
ECC: Eccentric
eNOS: Endothelial nitric oxide synthase
ERK1/2: Extracellular signal-regulated kinase ½
Ex: Exercise
F: Female
H^+^: Hydrogen ion
HIIE: High intensity interval exercise
HWI: Hot water immersion
KE: Knee extension
KF: Knee flexion
M: Male
MVIC: Maximal voluntary isometric contraction
PGC1A: Peroxisome proliferator-activated receptor gamma coactivator 1-alpha
P_i_: Inorganic phosphate
PLA: Placebo
Pmax: Maximal power obtained during a maximal incremental test
PO: Power output
PR: Passive recovery
Reps: Repetitions
RH: Relative humidity
RMS: Root mean square
RONS: Reactive oxygen and nitrogen species
SERCA: Sarcoendoplasmic reticulum calcium transport ATPase
SJ: Squat jump
SMVIC: Maximal voluntary isometric contraction with superimposed electrical stimulation
SR: Sarcoplasmic reticulum
RE: Resistance exercise
RT: Resistance training
TT: Time trial
TWI: Thermoneutral water immersion
TWP: Thermoneutral water immersion with placebo
VA: Voluntary activation assessed via interpolated twitch technique
VEGF: Vascular endothelial growth factor
Vmax: Maximal speed obtained during a maximal incremental test
VO_2_: Oxygen uptake
VO_2max_: Maximal oxygen uptake
VT: Ventilatory threshold
Wk: Week
Y: Year
